# Trans-Zeatin induce regulation the biosynthesis of 2-acetyl-1-pyrroline in fragrant rice (*Oryza sativa* L.) seedlings

**DOI:** 10.1186/s12870-023-04106-0

**Published:** 2023-02-11

**Authors:** Pipeng Xing, Haowen Luo, Zhenzhen He, Longxin He, Hua Zhao, Xiangru Tang, Meiyang Duan

**Affiliations:** 1 State Key Laboratory for Conservation and Utilization of Subtropical Agro-bioresources, College of Agriculture, South China Agricultural University, Guangzhou, 510642 China; 2 Scientific Observing and Experimental Station of Crop Cultivation in South China, Ministry of Agriculture and Rural Affairs, Guangzhou, 510642 China; 3 Guangzhou Key Laboratory for Science and Technology of Fragrant rice, Guangzhou, 510642 China; 4 Key Laboratory of Modern Biological Seed Industry in South China, Ministry of Agriculture and Rural Affairs, Guangzhou, 510000 China

**Keywords:** Gene expression, Trans-zeatin, Fragrant rice, 2-acetyl-1-pyrroline, Proline

## Abstract

**Background:**

In plants, cytokinin is activated into trans-zeatin to fight abiotic stresses. However, the mechanism of the effect of trans-zeatin on 2-acetyl-1-pyrroline (2-AP) biosynthesis in fragrant rice has yet to be studied. The present study was conducted to explore the effects of exogenous trans-zeatin on enzymes activities, genes expression, and precursors involved in 2-AP biosynthesis and 2-AP contents as well as the seedling quality of a fragrant rice cultivar viz., Meixiangzhan2. Four concentrations of trans-zeatin solutions at 20, 40, and 80 μmol L^− 1^ (ZT1, ZT2, and ZT3) were sprayed onto rice seedlings.

**Results:**

Compared to the control, trans-zeatin treatments showed significantly higher 2-AP contents of fragrant rice seedlings. Increased plant height and stem width were observed due to trans-zeatin treatments. The trans-zeatin application increased 1-pyrroline, methylglyoxal, proline, and P5C contents, enhanced P5CS and OAT activities, and reduced glutamic acid contents. In addition, expressions of *ProDH*, *P5CS2*, and *DAO4* were comparatively higher under trans-zeatin treatments than CK in fragrant rice seedlings.

**Conclusions:**

Overall, up-regulation of P5C, 1-pyrroline, and proline and down-regulation of glutamic acid under appropriate trans-zeatin concentrations (20 and 40 μmol L^− 1^) resulted in enhanced 2-AP biosynthesis in fragrant rice seedlings and 20–40 μmol L^− 1^ was considered as the suggested concentrations of trans-zeatin application in fragrant rice seedling.

## Background

Trans-zeatin, as a cytokinin derivative, is a central regulator of plant growth [1], which can help plants resist abiotic stress and regulate growth and development processes [2, 3]. Reports have shown that foliar spraying of exogenous cytokinins can also enhance the drought resistance of plants [4]. Arabidopsis seedlings improved their ability to resist low-temperature stress by applying exogenous cytokinins [5]. Nishiyama et al. [6] observed that exogenous trans-zeatin can enhance the antioxidant capacity of the tiny loquat seeds and modulate the level of endogenous trans-zeatin. However, the effects of trans-zeatin application on growth and 2-AP biosynthesis in fragrant rice have not been reported.

**Fig. 1:**
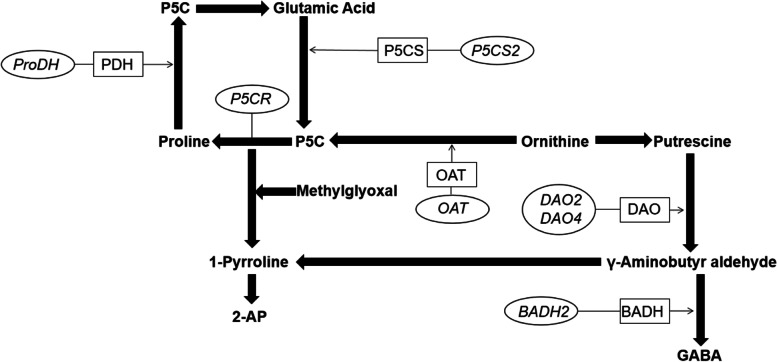
The schematic representation of 2-AP biosynthesis pathway in fragrant rice

Because of its unique aroma and excellent grain quality, fragrant rice is loved by consumers worldwide. Compared with nonfragrant rice, farmers can earn higher profits by planting fragrant rice [7]. Hundreds of compounds can contribute to the unique flavor of fragrant rice. Although hundreds of substances affect the aroma of fragrant rice, 2-AP is considered the most critical one [8, 9]. Figure 1 shows the known biosynthetic pathway of 2-AP in fragrant rice [10, 11]. It has been demonstrated that due to the *BADH2* gene silencing, 4-aminobutyraldehyde (GAB-ald) failed to be converted to 4-aminobutyric acid (GABA), resulting in GAB-ald accumulation, consequent activating biosynthesis of 2-AP in fragrant rice [12]. Research has shown that fragrant rice can accumulate more aromatic substances under abiotic stress, like heavy metals, salinity, and drought [13–15]. A possible explanation for this might be that BADH plays a role in abiotic stress tolerance in many plant species [16], and *BADH2* gene silencing promoted the accumulation of 1-pyrroline resulting in the synthesis of 2-AP in fragrant rice. Research shows that foliar application of paclobutrazol (a well-known plant growth regulator) could increase the 2-AP contents, improve grain quality, and enhance the productivity of fragrant rice. Despite numerous studies about fragrant rice, it is still being determined whether the phytohormone function is the biosynthesis of aroma compounds in fragrant rice.

Hence, we selected Meixiangzhan-2 as the material for the pot experiment, hoping to explain the mechanism of zeatin affecting 2-AP synthesis and further reveal the regulatory ability of zeatin as cytokinin on plant growth and development.

## Results

### 2-AP contents

**Fig. 2:**
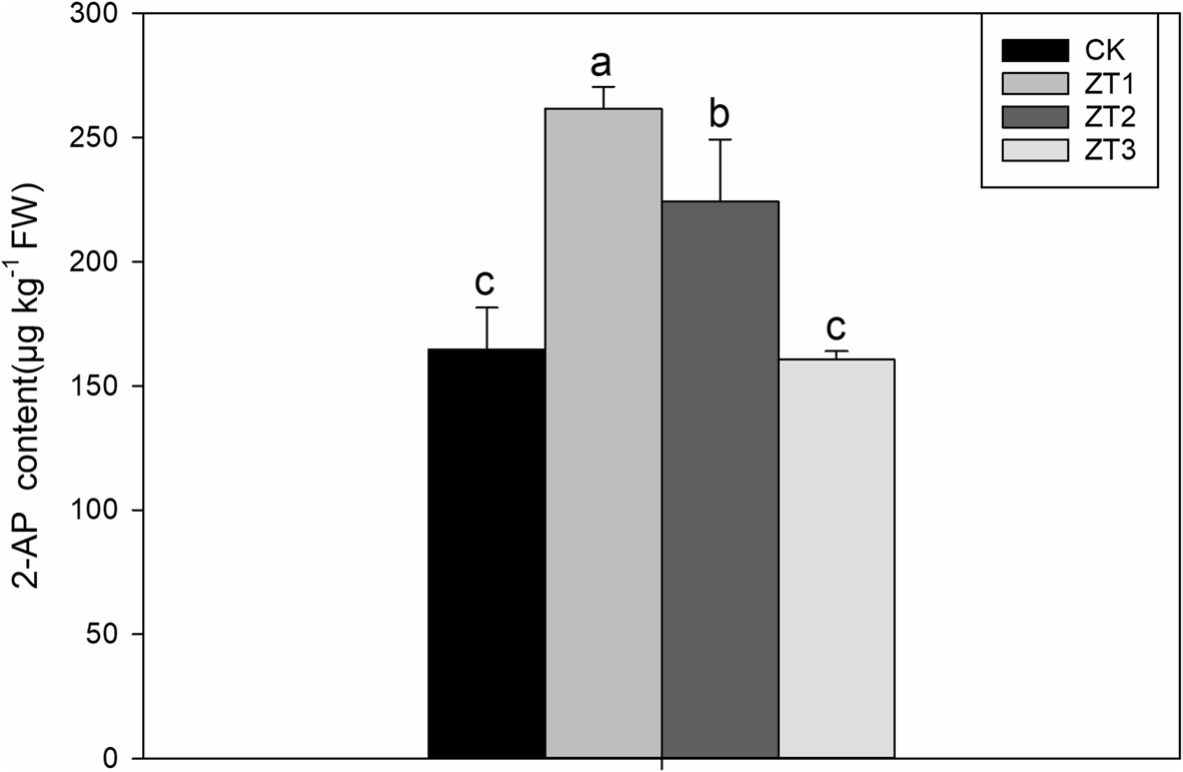
Effects of different trans-zeatin concentrations on 2-AP contents in fragrant rice seedlings. Each column represents the mean of three data±standard errors (*n* = 3). Bars sharing a typical letter do not differ significantly at *p* < 0.05

Different concentrations of trans-zeatin have significant effects on the 2-AP contents of plants (Fig. 2). ZT1 and ZT2 treatments significantly increased 2-AP contents by 59.10 and 36.58% compared with CK. Compared with CK, ZT3 treatment has no significant difference.

### Growth parameters

**Fig. 3:**
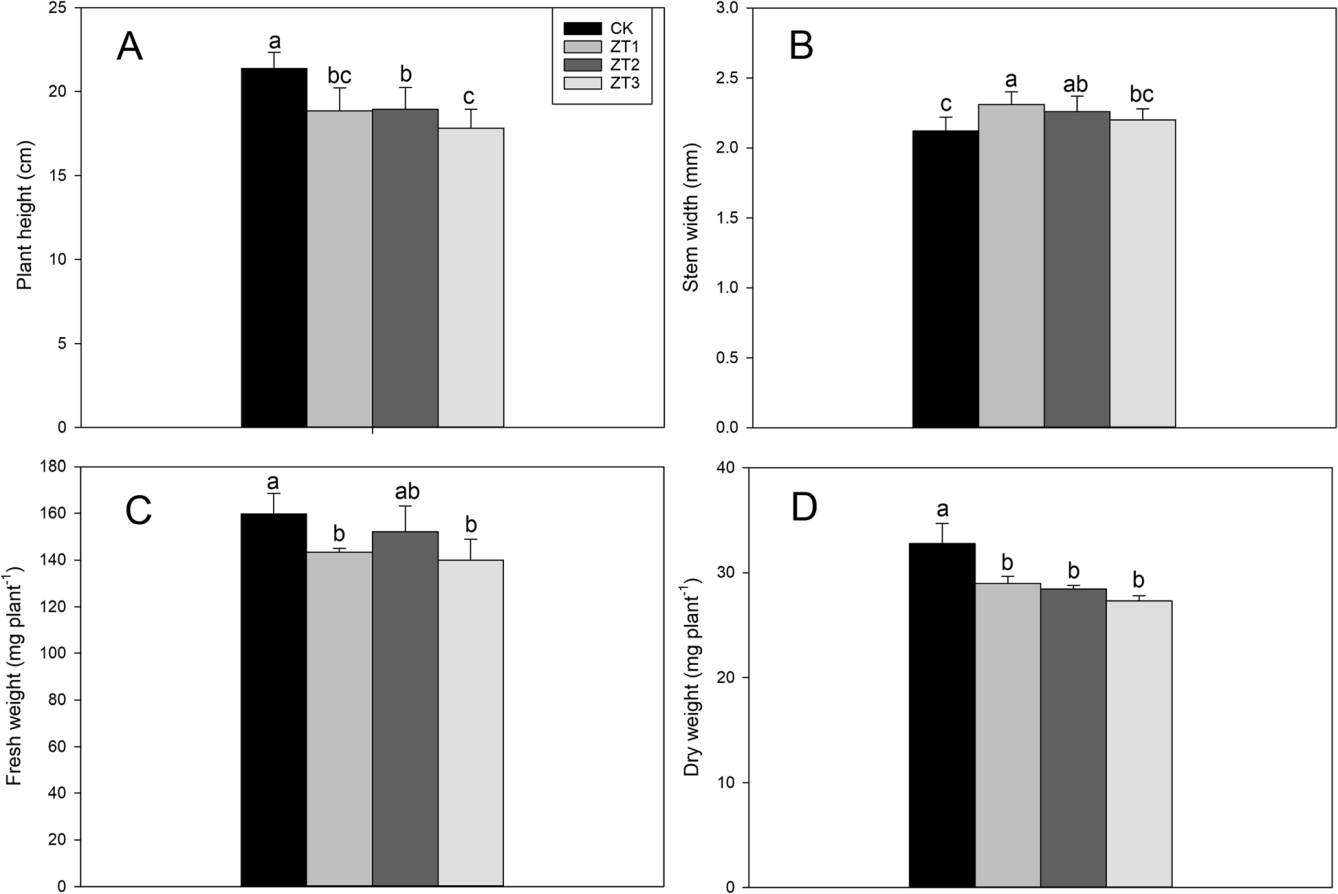
Effect of different trans-zeatin concentrations on on plant height (**A**), stem width (**B**), fresh weight (**C**) and dry weight (**D**) in fragrant rice seedlings. Each column represents the mean of three data±standard errors (*n* = 3). Bars sharing a typical letter do not differ significantly at *p* < 0.05

Figure 3 shows the effects of different trans-zeatin concentrations on fragrant rice growth parameters. ZT1, ZT2, and ZT3 treatments significantly decreased the plant height of plants by 11.78, 11.23, and 16.65% compared with CK, respectively. ZT1 and ZT2 significantly increased the stem width by 8.96 and 6.60%, respectively. ZT1 and ZT3 significantly decreased the fresh weight by 10.37 and 12.44%, respectively.

### 1-Pyrroline, methylglyoxal, glutamic acid, proline, GABA and P5C contents

**Fig. 4:**
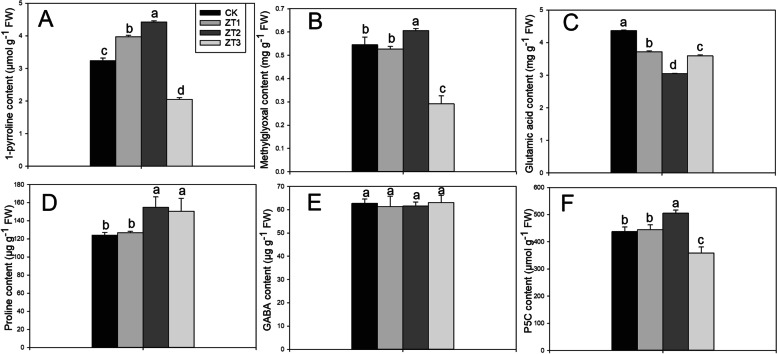
Effects of different trans-zeatin concentrations on 1-pyrroline (**A**), methylglyoxal (**B**), glutamic acid (**C**), proline (**D**), GABA (**E**), and P5C (**F**) contents in fragrant rice seedling. Each column represents the mean of three data±standard errors (*n* = 3). Bars sharing a typical letter do not differ significantly at *p* < 0.05

As shown in Fig. 4, 22.67 and 42.32% higher 1-pyrroline contents were recorded in ZT1 and ZT2 treatments than in CK, respectively. Higher methylglyoxal contents were recorded in ZT2 treatments compared with CK. The glutamic acid contents in ZT1, ZT2, and ZT3 treatments were lower than that in CK, but the proline contents in ZT2 and ZT3 treatments were higher than in CK. ZT2 treatments significantly increased P5C contents by 15.56%, but ZT3 treatments significantly decreased P5C contents by 18.08% compared with CK. The differences in GABA contents among ZT1, ZT2, ZT3, and CK treatments were insignificant.

### PDH, P5CS and OAT activities

**Fig. 5:**
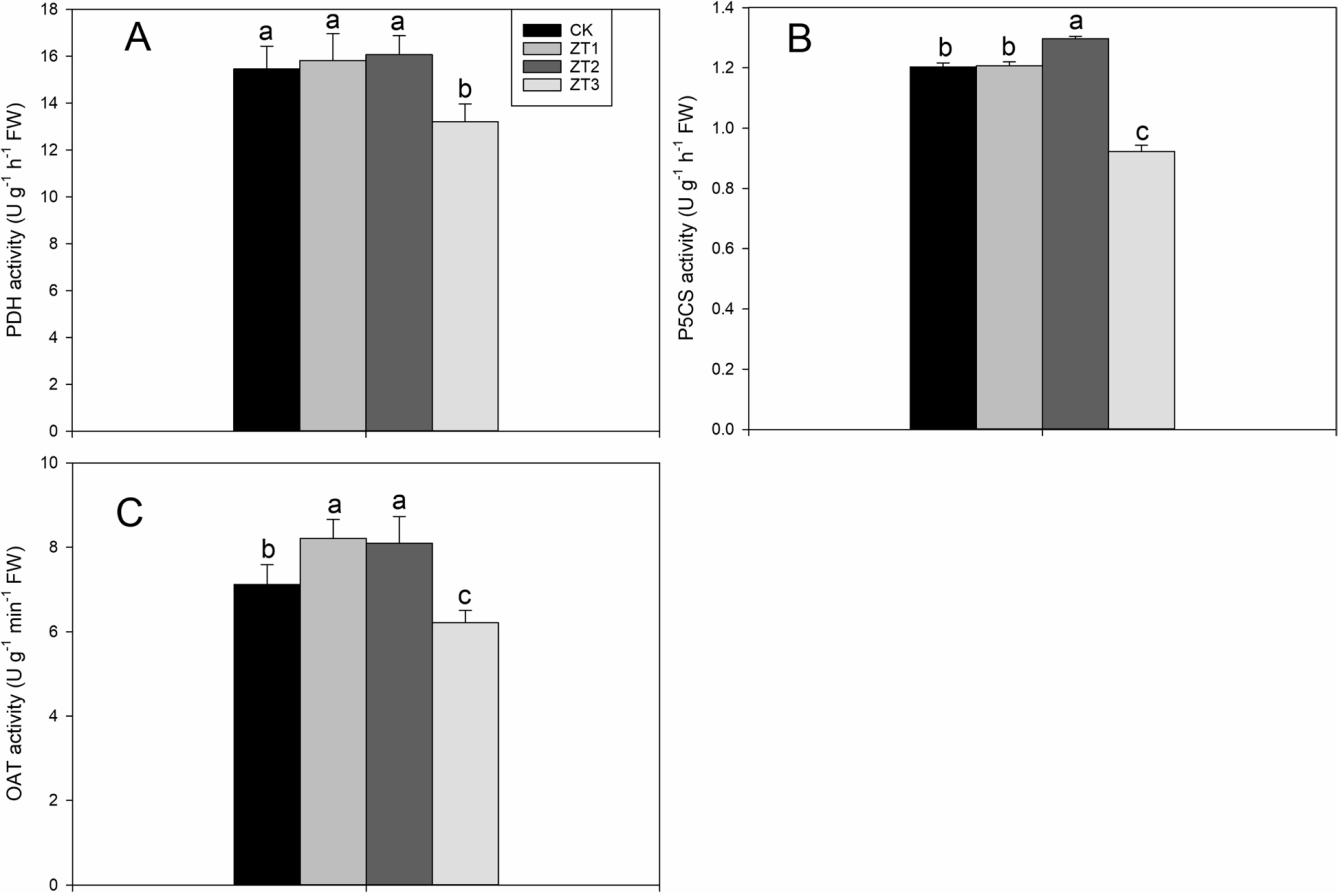
Effects of different trans-zeatin concentrations on activities of PDH (**A**), P5CS (**B**), and OAT (**C**) in fragrant rice seedlings. Each column represents the mean of three data±standard errors (*n* = 3). Bars sharing a typical letter do not differ significantly at *p* < 0.05

As shown in Fig. 5, the different concentrations of trans-zeatin can regulate PDH, OAT, and P5CS activities. Compared to CK, ZT3 treatment significantly decreased PDH activity by 14.55%. ZT2 treatments significantly increased P5CS activity by 7.85%, but ZT3 treatments significantly decreased P5CS activity by 23.36% compared with CK. Higher OAT activities were recorded in ZT1 and ZT2 treatments compared with CK.

### Expression levels of genes related to 2-AP biosynthesis

**Fig. 6:**
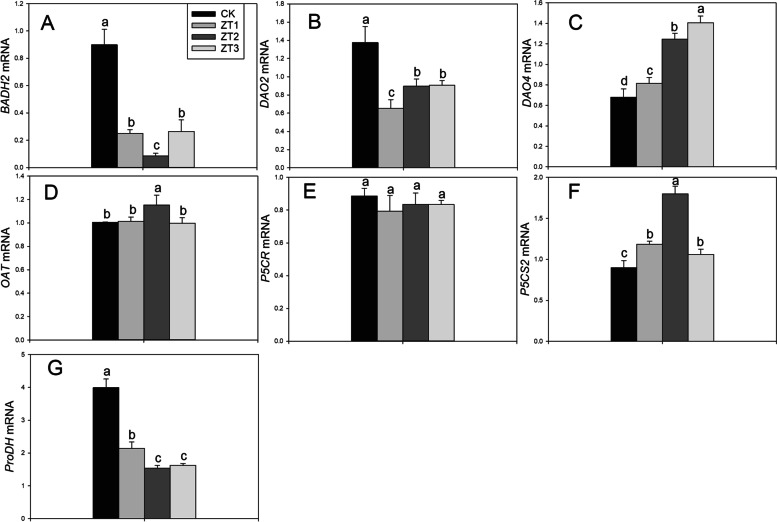
Effects of different trans-zeatin concentrations on expression levels of *BADH2* (**A**), *DAO2* (**B**), *DAO4* (**C**), *OAT* (**D**), *P5CR* (**E**), *P5CS2* (**F**), and *ProDH* (**G**) in fragrant rice seedling. Each column represents the mean of three data±standard errors (*n* = 9). Bars sharing a typical letter do not differ significantly at *p* < 0.05

Figure 6 shows the transcript levels of genes related to 2-AP under trans-zeatin concentrations. ZT1, ZT2, and ZT3 treatments significantly decreased transcript levels of *BADH2*, *DAO2*, and *ProDH* by 70.55–90.59%, 33.94–52.44%, and 46.39–61.44% compared with CK, respectively. The transcript level of *DAO4* increased with the addition of trans-zeatin concentrations. The transcription levels of *DAO4* in ZT1, ZT2, and ZT3 treatments were 20.19, 83.71, and 107.18% higher than that in CK, respectively. ZT2 treatment significantly increased transcript levels of *OAT*, but the differences in *OAT* among ZT1, ZT2, and CK treatments were insignificant compared with CK. As compared to CK, ZT2 treatment significantly increased the transcript level of *P5CS2* by 100.31%.

### Correlation analysis and principal component analysis

**Fig. 7:**
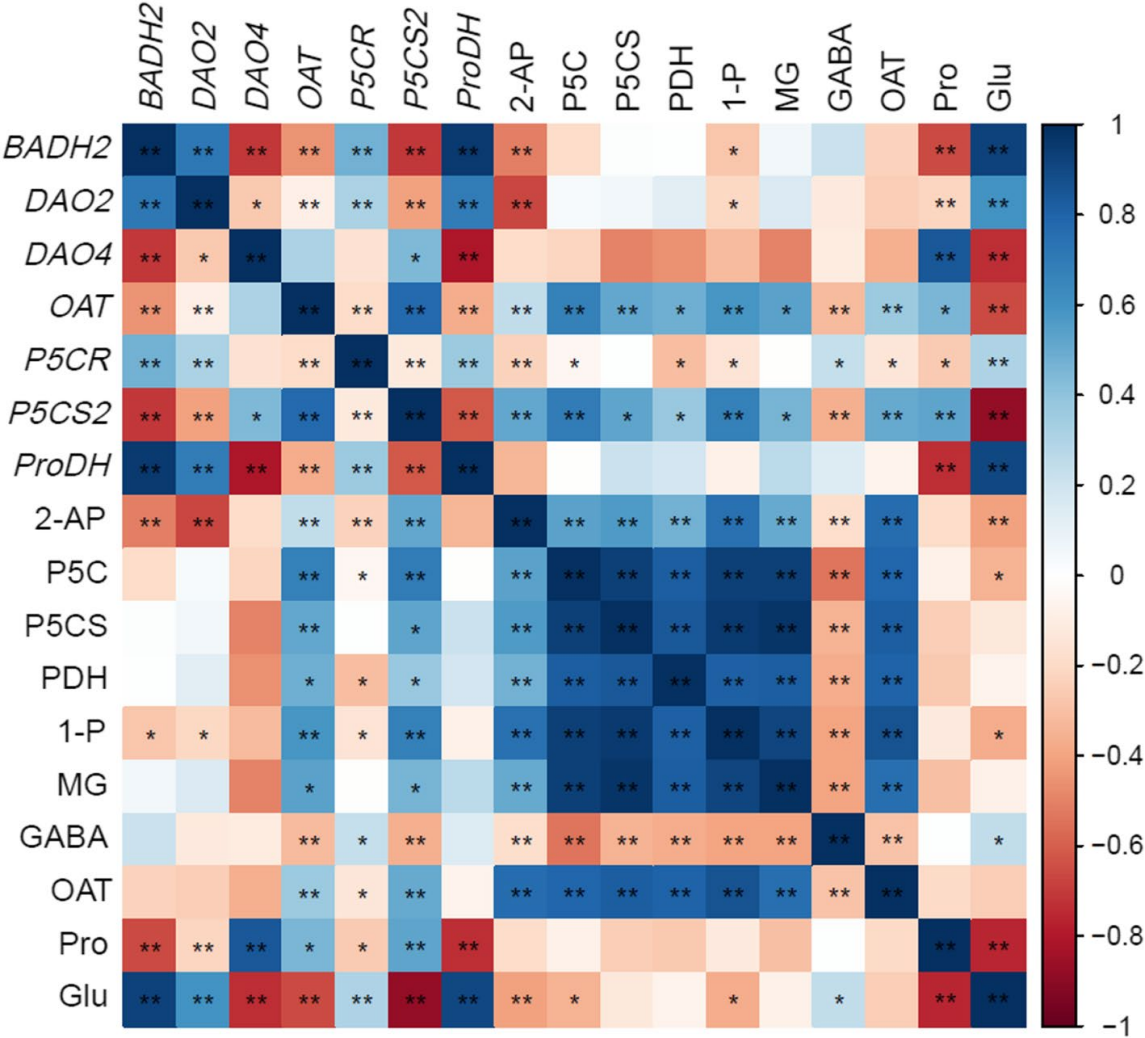
The heatmap for the investigated parameters. “*” show *p* < 0.05, and “**” show *p* < 0.01. 2-AP, 2-acetyl-1-pyrroline; 1-P, 1-pyrroline contents; MG, methylglyoxal contents; Glu, glutamic acid contents; P5C, pyrroline-5-carboxylic acid; PDH, proline dehydrogenase; P5CS, pyrroline-5-carboxylate synthetase; OAT, ornithine transaminase; Pro, Proline

The thermogram of correlations among enzyme activities, biochemical substances contents, gene expressions, and 2-AP contents is shown in Fig. 7. The contents of 2-AP in aromatic rice were negatively correlated with the transcription level of *BADH2*. 2-AP contents and P5CS2 transcriptional level were positively correlated with P5CS activity. Glutamic acid and GABA contents were negatively correlated with 2-AP contents, while proline contents were positively correlated with 2-AP contents. P5C, 1-pyrrolidine, and methylglyoxal contents were positively correlated with 2-AP, and the activities of PDH, P5CS, and OAT also showed a similar trend.

**Fig. 8:**
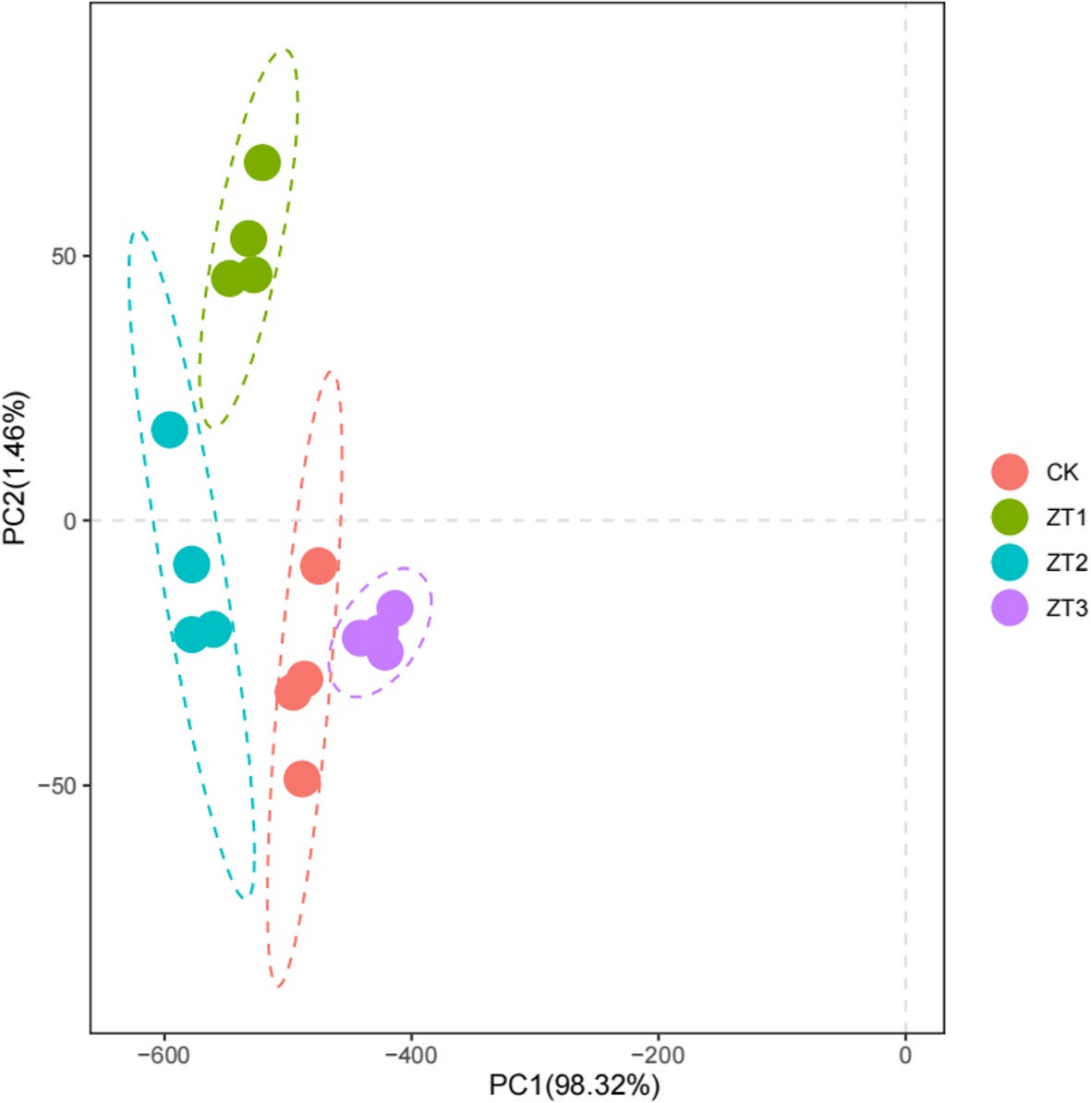
The principal component analysis plot for the investigated parameters

The principal component analysis plot of data from the enzymes activities, biochemical substances contents, genes expressions, and 2-AP contents is presented in Fig. 8. As shown in Fig. 8, the cumulative contribution of the first and the second principal components attained 99.78%. PC1 was highly contributed by P5C (− 0.8638), 2-AP (− 0.4032), and proline (− 0.2742). PC2 was mainly correlated to 2-AP (0.8883), proline (− 0.3431), and P5C (− 0.2940). The PC1 scores of ZT1 and ZT2 were much lower than CK as they had higher P5C, 2-AP, and proline contents. Compared with ZT1, the PC2 scores of ZT2 were much lower as it had higher contents of 2-AP.

## Discussion

There are hundreds of substances that affect the aroma of fragrant rice. 2-AP is considered to be the most critical one. Our results showed that appropriate trans-zeatin concentrations (ZT1 and ZT2) could increase the 2-AP contents of fragrant rice. The effect of trans-zeatin on the aroma of fragrant rice has not been reported. Our results also showed that appropriate trans-zeatin concentrations (ZT1 and ZT2) improved seedling quality by increasing stem width and decreasing plant height of fragrant rice seedlings. However, trans-zeatin treatment reduced fragrant rice seedlings’ fresh and dry weight.

Our study is consistent with that of Luo et al. [17]: appropriate concentrations of trans-zeatin can induce the accumulation of proline in fragrant rice, and the accumulation of proline promotes the synthesis of 2-AP. Proline was recognized as the precursor for 2AP biosynthesis [18]. Although PDH plays a crucial part in proline degradation [19, 20], its activity presented an opposite trend with increased proline contents. In contradiction to earlier findings [20], the increase in proline contents reduced the activity of PDH and the transcript level of *ProDH*. The decline in PDH activity could be attributed to transcript reduction. In addition, OAT and P5CS participate in the metabolic pathway of proline by converting ornithine and glutamic acid into proline [21, 22] and display higher activities under appropriate trans-zeatin concentrations (ZT1 and ZT2). In this experiment, meanwhile, contents of P5C, 1-pyrroline, and methylglyoxal had been increased as precursors and intermediates for the 2-AP biosynthesis. Real-time PCR analyses also showed that the expression of *OAT* and *P5CS2* increased inappropriate trans-zeatin concentrations (ZT1 and ZT2). From the results of the principal component analysis, compared with CK, ZT1 and ZT2 mainly have a gap on the PC1. P5C, 2-AP, and proline account for a large proportion of the PC1. Hence, we deduced that the increased expressions of *OAT* and *P5CS2* promote the increase of OAT and P5CS enzyme activities, thereby increasing the proline content.

Although glutamic acid and proline are the precursors of 2-AP [11, 23], proline is the most crucial precursor of aroma synthesis. Consistent with Woodrow et al. [24], our study shows that proline contents negatively correlate with glutamate under certain conditions. The P5CS and its encoded gene (*P5CS2*) exhibited higher activity and transcript levels under appropriate trans-zeatin concentrations. It showed that appropriate trans-zeatin concentrations significantly decreased glutamic acid by promoting its degradation. We deduced that the proline accumulation induced the rise of P5CS and OAT activity and gene expression, thus causing glutamic acid accumulation. 2-AP can also be biosynthesis by converting ornithine into putrescine, and it is ultimately biosynthesis from 1-pyrroline and an acetyl group or methylglyoxal via the non-functional *BADH2* [25–27]. Though Our study noted that the transcript level of *DAO4* was greatly improved, the transcript level of *DAO2* presented decreased trends with the trans-zeatin application. In correlation analysis, the transcript level of *DAO4* was no significant relationship with the 2-AP contents.

Our study observed that appropriate trans-zeatin concentrations (ZT2 and ZT3) induced proline accumulation. In addition to being the precursor for the 2-AP biosynthesis [18], proline also plays multiple roles in plant physiological activities such as stress responses [20, 28]. Therefore, we deduced that trans-zeatin increased the 2-AP contents of fragrant rice seedlings because it activated the stress resistance mechanism of rice and promoted the synthesis and accumulation of proline and its precursors. Cytokinins also play a fundamental role in establishing source-sink relationships among plant tissues and organs [29]. However, it is still being determined how 2-AP and its precursors would be transported in fragrant rice plants. Therefore, it is necessary to study further the biosynthesis of 2-AP and its response to trans zeatin.

## Conclusion

This study shows that at appropriate trans-zeatin concentrations (20 and 40 μmol L^− 1^), the increase of 2-AP contents in fragrant rice is mainly through the up-regulated expressions of *P5CS2*, promoting the transformation of proline to 2-AP. At the same time, the glutamate and ornithine pathways were inhibited. Trans-zeatin also increased the content of aroma synthesis precursor 1-pyrroline and methylglyoxal and improved the quality of fragrant rice seedlings.

## Methods

### Plant materials, growth condition, and experimental design

The pot experiment was conducted at the College of Agriculture, South China Agricultural University, Guangzhou, China, during October and November 2021. The fragrant rice variety Meixiangzhan-2, widely planted in Guangdong, was selected as the plant material in the experiment. Rice materials are provided by the Rice Cultivation Research Office, College of Agriculture, South China Agricultural University. Detailed information on the rice variety can be found at https://www.ricedata.cn/variety/. Before sowing, the seeds were soaked in distilled water for 12 h, taken out, washed with distilled water, wrapped with a wet towel, put into the thermostat, and transferred to a constant temperature (36 °C) for 12 h. After germination, the seeds were sown into pots containing 10 kg of paddy soil(containing 20.01 g kg^− 1^ organic matter, 1.009 g kg^− 1^ total nitrogen, 21.04 g kg^− 1^ total potassium, 1.101 g kg^− 1^ total phosphorus, 105.11 mg kg^− 1^ alkali-hydrolyzable nitrogen, and 60.03 mg kg^− 1^ available potassium, 120.34 mg kg^− 1^ available phosphate, 6.16 pH). The rice seedlings were grown under controlled conditions in an artificial climate box (25 °C, 60% relative humidity, and 100 μmol m^− 2^ s^− 1^ for 16 h and dark for 8 h). Fourteen-day-old seedlings were subjected to four concentrations of trans-zeatin solutions treatments (20 μmol L^− 1^ (ZT1), 40 μmol L^− 1^ (ZT2), and 80 μmol L^− 1^ (ZT3)), and 10 pots for each treatment. The treatment applied with distilled water was set as the control (CK). The trans-zeatin (C_10_H_13_N_5_O, CAS No.1637-39-4) used in the experiment was produced by Shanghai yuanye Bio-Technology Co., Ltd. Refer to Luo et al. [30] for sample processing method 5 days after receiving the treatments, the plants of rice seedlings were all sampled for biochemical and molecular determination, and were stored at − 80 °C. At the same time, 50 seedlings with similar growth were selected from each treatment for stem diameter, fresh weight, and plant height determination. After measuring the fresh weight of the plant from each treatment, the plants were oven-dried at 105 °C for 15 min and then at 60 °C for 48 h to determine dry weight.

### Determination of 2-AP, 1-pyrroline, proline, methylglyoxal, GABA, and P5C contents

The 2-AP contents were determined with synchronization distillation and extraction method (SDE) combined with GCMS-QP 2010 Plus (Shimadzu Corporation, Japan) according to the methods of Luo et al. [17]. The final 2-AP contents were expressed as μg kg^− 1^ fresh weight (FW). The determination of 1-pyrroline contents was carried out with the methods of Hill [31]. The 1-pyrroline concentrations were calculated with the molar extinction coefficient (ε = 1860 cm^-l^) after the absorbance was read at 430 nm. The final 1-pyrroline contents were expressed as μmol g^− 1^ FW. The determination of proline contents was carried out with the method of Bates et al. [32]. The absorbance was measured at 520 nm, and the concentrations were calculated from a standard curve. The final proline contents were expressed as μg g^− 1^ FW. The determination of methylglyoxal contents was carried out [33]. The absorbance of the derivative was read at 336 nm, and the concentrations were calculated from a standard curve. The final methylglyoxal contents were expressed as mg g^− 1^ FW. The determination of GABA contents was carried out with the methods of Bao et al. [33]. The absorbance was measured at 645 nm, and the concentrations were calculated from a standard curve. The final GABA contents were expressed as μg g^− 1^ FW. The determination of P5C contents was carried out with the methods of Bao et al. [33]. The P5C concentrations were calculated with the molar extinction coefficient (ε = 2.58 mM cm^− 1^) after the absorbance was read at 430 nm. The final P5C contents were expressed as μmol g^− 1^ FW. The determination of each sample consists of three replicates.

### Determination of P5CS, PDH, and ornithine transaminase activities

P5CS activity was determined according to the methods of Sánchez et al. [34]. The absorbance was read at 535 nm after the reaction. The determination of PDH activity was carried out according to Ncube et al. [35] methods, and the absorbance was read at 440 nm after the reaction. The determination of OAT activity was carried out according to the methods of Deng et al. [36], and the absorbance was read at 440 nm. The determination of each sample consists of three replicates.

### Real-time quantitative RT-PCR

Real-time quantitative method refers to Luo et al. [30], and the specific method is as follows: Total RNA was extracted using the HiPure Plant RNA Mini Kit (Magen, Guangzhou, China). The quality and quantity of RNA were assessed by NanoDrop 2000. The Hiscript II QRT SuperMix for qPCR (+gDNAwiper; Vazyme, Nanjing, China) synthesized cDNA from total RNA. Real-time quantitative RT-PCR (qRT-PCR) was conducted in the CFX96 real-time PCR System (Bio-Rad, Hercules, CA, United States). Actin was used as an internal reference gene. Each RNA sample was performed in triplicate. A negative control without a cDNA template was always included. Primers used for qRT-PCR are listed in Table 1. All primers were designed using the software tool Primer 5 (Premier Biosoft International, Palo Alto, CA).

**Table 1:** Primer sequences of genes encoding enzymes involved in 2-AP biosynthesis

Gene name	Accession no.	Primer sequences
Proline dehydrogenase (PRODH)	AP014966.1	F 5′-TCATCAGACGAGCAGAGGAGAACAGG-3’
		R 5′-CCCAGCATTGCAGCCTTGAACC-3’
Pyrroline-5-carboxylic acid synthetase2 (P5CS2)	AP014957.1	F 5′-GAGGTTGGCATAAGCACAG-3’
		R 5′-CTCCCTTGTCGCCGTTC-3’
Ornithine aminotransferase (OAT)	AP014959.1	F 5′-GCCCTTGGTGCTGGAGTA-3’
		R 5′-AGCCCTTTCAACGAGACCTT-3’
Diamine oxidase2 (DAO2)	AP014960.1	F 5′-TCGTTCGCATCAAGGTTGG-3’
		R 5′-TCAGACAGAAGGGTGCCGTA-3’
Diamine oxidase4 (DAO4)	AP014960.1	F 5′-TGGCAAGATAGAAGCAGAAGT-3’
		R 5′-GTCCATACGGGCAACAAA-3’
Betaine aldehyde dehydrogenase (BADH2)	AB09683	F 5′-GGTTGGTCTTCCTTCAGGTGTGC-3’
		R 5′-CATCAACATCATCAAACACCACTAT-3’
OsActin	AK101613	F 5′-CTTCATAGGAATGGAAGCTGCGGGTA-3’
		R 5′-CGACCACCTTGATCTTCATGCTGCTA-3’

### Data analysis

All the obtained data were subjected to a one-way analysis of variance (ANOVA) using R 4.1.2. R 4.1.2 was also used to perform correlation analysis and principal component analysis (PCA), and the figure for the investigated parameters was established. The differences among means were separated using the least significant (LSD) test at the 5% probability level. Sigma Plot 13.0 (Systat Software Inc., San Jose, CA, United States) was used to make figures.

## Data Availability

The original contributions presented in the study are included in the article/Supplementary Material, and further inquiries can be directed to the corresponding author.

## Abbreviations

2-AP: 2-acetyl-1-pyrroline
P5CS: 1-pyrroline-5-carboxylate synthetase
GAB-ald: 4-aminobutyraldehyde
GABA: γ-aminobutyric acid
PDH: Proline dehydrogenase
P5C: pyrroline-5-carboxylic acid
BADH: Betaine aldehyde dehydrogenase
OAT: Ornithine transaminase
DAO: Diamine oxidase
